# Bioactive Cellulose Acetate Electrospun Mats as Scaffolds for Bone Tissue Regeneration

**DOI:** 10.1155/2022/3255039

**Published:** 2022-02-04

**Authors:** Simara Laboy-López, Pedro O. Méndez Fernández, Jorge G. Padilla-Zayas, Eduardo Nicolau

**Affiliations:** ^1^Department of Chemistry, University of Puerto Rico, Rio Piedras Campus, 17 University Ave. 1701, San Juan 00925, USA; ^2^Molecular Science Research Center, University of Puerto Rico, 1390 Ponce De Leon Ave, Suite 2, San Juan 00931-3346, USA; ^3^Department of Biology, University of Puerto Rico, Rio Piedras Campus, PO Box 23346, San Juan 00931-3346, USA

## Abstract

In the last decades, cell-based approaches for bone tissue engineering (BTE) have relied on using models that cannot replicate the complexity of the bone microenvironment. There is an ongoing amount of research on scaffold development responding to the need for feasible materials that can mimic the bone extracellular matrix (ECM) and aid bone tissue regeneration (BTR). In this work, a porous cellulose acetate (CA) fiber mat was developed using the electrospinning technique and the mats were chemically modified to bioactivate their surface and promote osteoconduction and osteoinduction. The mats were characterized using FTIR and SEM/EDS to validate the chemical modifications and assess their structural integrity. By coupling adhesive peptides KRSR, RGD, and growth factor BMP-2, the fiber mats were bioactivated, and their induced biological responses were evaluated by employing immunocytochemical (ICC) techniques to study the adhesion, proliferation, and differentiation of premature osteoblast cells (hFOB 1.19). The biological assessment revealed that at short culturing periods of 48 hours and 7 days, the presence of the peptides was significant for proliferation and adhesion, whereas at longer culture times of 14 days, it had no significant effect on differentiation and maturation of the osteogenic progenitor cells. Based on the obtained results, it is thus concluded that the CA porous fiber mats provide a promising surface morphology that is both biocompatible and can be rendered bioactive upon the addition of osteogenic peptides to favor osteoconduction leading to new tissue formation.

## 1. Introduction

Bone defects have been traditionally treated with autologous and allogeneic bone implants. However, donor site morbidity, host immune rejection, and shortage of allogeneic bone for grafts limit these therapeutic approaches. Bone tissue analogues developed applying the principles of tissue engineering constitute a novel therapeutic alternative to treat craniofacial bone defects. Bone tissue analogues are made with scaffolds that simulate the extracellular matrix (ECM) composition and structure. The ideal scaffold must have several characteristics; among them are biocompatibility, porosity, and mechanical strength. The bone is a porous structure with interconnected channels that allow the development of blood vessels and nerves. Besides pore interconnectivity, scaffolds should be three-dimensional structures possessing high surface area and roughness. This allows for cell-biomaterial interactions leading to adhesion and extracellular matrix (ECM) deposition for the development of mature osteoblasts that will deposit the bone mineral (i.e., hydroxyapatite) to form new bone and the collagen matrix [1]. Also, the fabrication of semirigid scaffolds (i.e., mechanical strength) that are flexible provides many advantages as it allows for alteration and implantation with minimal effort [2]. Nevertheless, research has shown that scaffold fabrication has been somewhat limited by the sacrifice between porosity and mechanical strength [3].

Osteoblasts are anchorage-dependent cells that respond to chemical and mechanical stimuli for cell movement and adhesion. Cell adhesion is promoted by the presence of integrins, which are transmembrane heterodimeric receptors consisting of noncovalently associated alpha (*α*) and beta (*β*) subunits [4]. Osteoblast cells have various integrin receptors, and a recent study by Olivares-Navarrete et al. revealed the importance of the *β*1 subunit for roughness recognition and the *α* subunits for chemical surface recognition [5]. The KRSR peptide sequence that binds to heparan sulfate (HS) has been found in different bone-related adhesive proteins and has shown to be selective for osteoblast adhesion. In a study evaluating the cellular adhesion from different cell types, it was found that the addition of a heparin-binding peptide was required for proliferation and anchoring to the substrate since HS is a principal component of glycosaminoglycans [6, 7]. Besides glycosaminoglycans, osteoblasts recognize arginine-glycine-aspartic acid-X (RGD) sequences for adhesion and proliferation. These are adhesive peptide sequences, which mediate integrin receptor interactions and are exhibited by many ECM molecules such as fibronectin, vitronectin, bone sialoprotein, and osteopontin. In fibronectin, a type of adhesive glycoprotein, RGDX recognizes a number of integrin ligand receptors like *α*_v_*β*_3_ ligands on various cell types [8]. The *α*_v_*β*_3_ integrin is highly expressed in osteoblast cell lines as its osteoinductive properties were shown by Fraioli et al. and resulted in stimulating significantly cell adhesion [9]. Also, bone morphogenetic protein-2 has been widely studied as an osteoinductive protein and has been regarded as the most potent. However, the complex multilevel structure of BMPs makes it prone to degradation. This downside has led researchers to isolate different peptide sequences that could promote osteoblast adhesion and maturation [10]. Specifically, it was found that the peptide sequence KIPKASSVPTELSAISTLYL is responsible for bone cell proliferation and differentiation [11]. In this work, we take advantage of previously known osteoblast bioactive-promoting peptides to enhance pre-osteoblast adhesion and maturation on functionalized cellulose acetate (CA) fiber mats.

The CA fiber mats were developed using the electrospinning (ES) technique. This technique generates nanometric to micrometric fibers that resemble the ECM and enhance surface area for osteoblast adhesion [12, 13]. These properties counter the ones observed over the years with other techniques that fail to reproduce either the complexity of the bone 3D microenvironment or the nanoscale characteristics of mineralized bone collagen [14, 15]. To create our scaffolds, we utilized cellulose acetate (CA) because it has been proven to be biocompatible and biodegradable, and possesses excellent mechanical properties [16, 17]. Electrospun fibers have been found to possess the physical requirements for bone tissue regeneration, but many researchers have also added bioactive agents to make the fibers bioactive [18]. Due to this, we modified the CA fiber mats covalently with different osteoblasts adhesive peptides to promote bioconductivity and study their potential to be used as a scaffold material in the field of bone tissue regeneration [19]. Covalently linked peptides could enhance the interaction between osteoblastic cell types and the scaffold and could provide a long-lasting binding site for bone tissue regeneration. This study evaluates the osteoconductive and osteoinductive properties of bioactive CA fiber mats with pre-osteoblastic cell lines for the purpose of providing an ideal scaffold to aid in bone tissue regeneration.

### 1.1. Materials

All reagents and solvents used in the next experiments were analytical reagent grade and utilized without additional purification or processing. Cellulose acetate (CA) with an average molecular weight (Mn) of 30,000 was purchased from Sigma-Aldrich (USA). Acetone for HPLC and chloroform (anhydrous) were purchased from Sigma-Aldrich (USA) and used as solvents for CA. Materials purchased for electrospinning were used as received. The in vitro bioactivity of the developed scaffolds was tested using human fetal osteoblastic cell line hFOB 1.19 from ATCC® CRL-11372™. Dulbecco's modified eagle's medium (DMEM)/Nutrient mixture F-12, G418 Geneticin, and fetal bovine serum (FBS) were purchased from Gibco-Thermo Fisher Scientific. Trypsin-EDTA (0.05%–0.5 mM) was procured from Sigma-Aldrich (USA). Nanopure water (18.2 MΩ cm^2^, Milli-Q Direct 16) was always used.

### 1.2. Fabrication

Following a previous report by Tungprapa et al. [20] on the morphological effect of solvent systems with CA electrospun fibers, a 5% CA solution was prepared by dissolving a certain amount of the polymer (CA, 30,000 g/mol) in a 9 : 1 mixture of CCl_4_ and acetone. The solution was left in constant stirring for a period of 20 hours. Then, the solution was used to fill a conductive syringe connected to the positive electrode of the high voltage power supply (Gamma High Voltage Research). The needle was kept 10 cm apart from the collector with a delivery flow rate of 3 mL/h under a fixed voltage of 7 kV at 1000 rpm. The fiber mats were stripped from the aluminum foil by submersion in methanol followed by thorough washing using Nanopure water.

The methods most used to attach peptides to a substrate are adsorption, silanization, click chemistry, and carbodiimide crosslinking. [21] The latter was chosen because it does not involve the addition of other atoms to the final structure and the reaction is performed under mild and aqueous conditions, thereby reducing the risk of fiber degradation. As a strategy to activate the cellulose acetate fibers and prepare them for chemical conjugation with the bioactive peptides, surface modifications were performed. First, the fiber mats were deacetylated following well-established procedures published elsewhere [22]. The degree of deacetylation was assessed using FTIR from 5–30 min and 1–3 h. This process was optimized, and a period of 10 minutes was selected for deacetylation using 0.2 M NaOH in 95% EtOH. The fiber mats were thoroughly washed with water before further use. Then, the fibers were oxidized to allow the formation of carboxylic terminals for further bioconjugation. [23] Following a previously reported procedure, the oxidation was performed using a mixture of sodium hypochlorite (NaOCl), sodium bromide (NaBr), and 2,2,6,6-tetramethyl-1-piperidinyloxy radical (TEMPO) to selectively oxidize the primary hydroxyl groups that resulted from the previous deacetylation procedure. [24] In brief, the fibers were oxidized by mixing 64.8 mg of NaBr and 59.0 mg of TEMPO in 100 mL of H_2_O along with ≈20 mg of fiber mat. Then, 2 mL of a 12% NaOCl was added and the pH was fixed to 10. Afterwards, the solution was left to react for 1 h on a shaker and the reaction was stopped by adding 20 mL of methanol and adjusting the pH to 7. The fiber mats were removed from the solution and washed thoroughly with water.

**Scheme 1:**
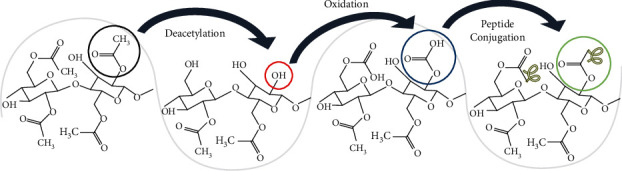
Representation of the CA fiber mat activation and modifications to covalently attach the bioactive peptides.

Thereafter, the carbodiimide crosslinking technique was utilized to further activate the carboxylic acids to attach the selected peptides. The carbodiimide used was 3-(dimethylaminopropyl)-3-ethylcarbodiimide (EDC) with sulfo-N-hydroxysuccinimide (sulfo-NHS) to conjugate the peptides KRSR (≈12 mg), RGD (≈8 mg) and BMP-2 (≈7 mg) to the oxidized fiber mats. The peptides were purchased from GenScript with 3 aminohexanoic acid units added to each sequence at the N terminus and the observed molecular weight of KRSR = 885.2, RGD = 830.0, and BMP-2 = 2457.6 as provided by the supplier; a representation of the structures is shown in Table S1. In brief, 18 mg of the oxidized fiber mat was allowed to react with 14.4 mg of EDC, 19.8 mg of sulfo-NHS, and 50 mL of Milli-Q Nanopure water at a neutral pH. Then, the product was thoroughly washed and stored until further use. A summary of these modifications is shown in Scheme 1.

### 1.3. Scaffold Characterization

After room temperature drying, the morphology and chemical modifications of the fiber mats were assessed. Scanning electron microscopy (SEM) images were recorded using a JEOL JSM-6480LV Scanning Electron Microscope with a electron beam energy of 20 kV. The fiber mats were coated with a thin gold film (12 nm), and several images were collected at a 5,500 magnification.

Fourier transform infrared spectroscopy (FTIR) spectrums were obtained by using a Bruker Tensor 27 spectrometer using the attenuated total reflectance mode. The spectra were recorded between 500 and 4000 cm^−1^, with a 4 cm^−1^ resolution and with a total of 300 scans.

### 1.4. Biocompatibility Studies

**Scheme 2:**
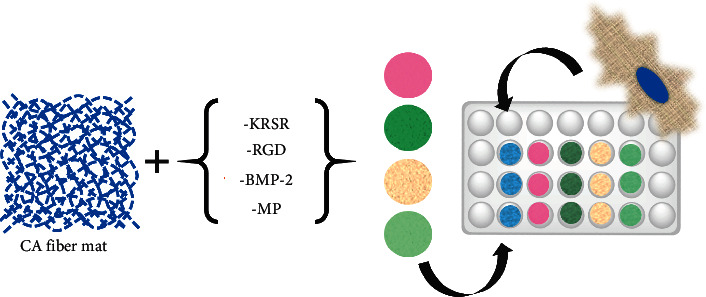
Experimental well plate design for immunocytochemical analysis.

Fetal osteoblasts, hFOB 1.19 (ATCC CRL-11372), were cultured in 75 cm^2^ tissue culture flasks and utilized for the biological characterization of the materials. The culture medium used was a 1 : 1 mixture of Ham's F12 medium Dulbecco's modified Eagle's medium, with 2.5 mM L-glutamine with added 0.3 mg/ml G418 and fetal bovine serum to a final concentration of 10%. The control well plates were designed (Scheme S1) with a positive and negative untreated glass disc, and triplicates of CA fiber mat with peptides dissolved in culture medium as secondary controls (Figures S6–9). An experimental well plate was designed with CA fiber mat and peptide-conjugated fiber mats in triplicate as shown in Scheme 2. All cellular staining and fluorescence images were observed and analyzed with a Nikon Eclipse Ti-E Inverted A1 Confocal Microscope. For each fiber mat sample, three spots were pictured using three fluorescence channels for DAPI, Phalloidin, and Alexa Fluor 488 Fluorescent labels. All metadata is stored in the original image files available upon request.

#### 1.4.1. Cell Adhesion

Cells were seeded in a 24-well plate at 1 × 10^5^ cells per well in contact with the fiber mats (1/2” diameter) and cultured for a period of 7 days replacing the culture medium every other day. First, the cells were fixed with paraformaldehyde (4% in PBS) and rinsed in triplicate with PBS after 15 mins. Following this step, the cells were permeabilized with Triton-X100 (0.1% in PBS) for 15 mins and washed with PBS again. To block antibody binding to nonspecific sites, a goat serum (2% in PBS with sodium azide, NaN_3_) solution was added for 1 h. Upon removal, the cells were incubated with Anti-Integrin alpha V beta 3 antibody [BV3] (1 : 500) and placed overnight in a wet chamber at 4°C. After incubation with the primary antibody, the cells were washed with PBS a total of 4 times and 10 minutes and subsequently incubated in dark for 1 h with Polyclonal Alexa Fluor 488 AffiniPure Goat Anti-Mouse IgG (*H* + *L*) secondary antibody (1 : 500, Jackson Immuno Research) and Phalloidin-iFluor 647 Reagent (1 : 500, Abcam). Lastly, the samples were washed 4 times with PBS for 10 mins and mounted on 24 × 60 mm coverslips with ProLong Diamond Antifade reagent containing 4′, 6-diamidino-2-phenylindole (DAPI) (Abcam) and left in dark at room temperature until microscope analysis.

#### 1.4.2. Cell Morphology

The morphology of the cells was studied by integrating Phalloidin 647 iFluor Reagent together with the secondary antibody used when performing the immunocytochemical procedures. A complimentary technique used in this assessment was SEM. To study the morphology of the cells by SEM, after a 14-day incubation period, the cells were fixed with glutaraldehyde and stored at 4°C overnight. After removing the glutaraldehyde, they were rinsed three times with PBS for 10 min. The fiber mats were washed in a series of EtOH solutions beginning at 10%, 20%, all the way up to 90% for a period of 10 min each and finishing with three washes at 100%. The cells were chemically dehydrated by HMDS by rinsing in pure HMDS/EtOH ratios of 1 : 3, 1 : 1, and 3 : 1 for periods of 10 min and finishing with triple HMDS washes and overnight evaporation of HMDS-filled well. They were gold-coated (12 nm) and examined at x500 and x2500 magnifications using a High-Resolution JSM-IT500HR Scanning Electron Microscope with a electron beam energy of 20 kV.

#### 1.4.3. Cell Proliferation

Cellular adhesion was also assessed by counting the number of cells present in the fiber mats. This was performed using 5-bromo-2′-deoxyuridine, BrdU (B5002 Sigma). Cells were seeded in a 24-well plate at 50,000 cells per well in contact with the fiber mats for 48 hours. After incubation, DMEM was removed from each well and a 10 *μ*M BrdU solution in DMEM was added and incubated for 6 h. The BrdU solution was removed from each well, and the fiber mats were rinsed with PBS for 1 min. Afterwards, the cells were fixed with paraformaldehyde (4% in PBS) and rinsed in triplicate with PBS after 15 mins. Then, the cells were permeabilized with Triton-X100 (0.1% in PBS) for 15 mins and washed with PBS again. Following this step, for the purpose of denaturing the DNA, HCl at 0.05 N was added for 1 hour and rinsed with PBS for 5 minutes. A goat serum (2% in PBS/NaN_3_) solution was added for 1 h to block antibody binding to nonspecific sites, and when removed, the cells were incubated with anti-BrdU solution (1 : 5) and placed overnight in a wet chamber at 4°C. The next day, the solution was removed from the well plates and the fiber mats were rinsed 3 times with PBS for 10 minutes. After rinsing, polyclonal Alexa Fluor 594 AffiniPure Goat Anti-Mouse IgG (*H* + *L*) secondary antibody (1 : 500, Jackson Immuno Research) was added and left in dark at room temperature for 1 h. Lastly, the samples were washed 3 times with PBS for 10 mins and mounted on 24 × 60 mm coverslips with ProLong Diamond Antifade reagent containing 4′, 6-diamidino-2-phenylindole (DAPI) (Abcam) and left in dark at room temperature until microscope analysis.

#### 1.4.4. hFOB Differentiation and ECM Formation on Mats

Maturation assays were performed following the same previously used methodology used to study cell adhesion. For these specific assays, 75,000 cells were seeded per well to avoid overlapping fluorescence and the culture medium was replaced every other day until fixation. The immunocytochemical procedure was performed with the same fixation and staining protocol used for the *α*_V_*β*_3_ integrin with the distinction of the primary antibody used. For the collagen expression assessment, the corresponding antibodies used were anti-collagen I antibody (ab34710) and secondary Alexa Fluor 488 Goat Anti-Mouse IgG (*H* + *L*) at (1 : 500) with Phalloidin-iFluor Reagent 647 (1 : 500). And, the antibodies employed for BSP expression were primary antibody Anti-Bone Sialoprotein antibody (ab52128) and secondary Alexa Fluor 488 Goat Anti-Rabbit IgG (*H* + *L*) at (1 : 500) with Phalloidin-iFluor Reagent 647 (1 : 500).

### 1.5. Statistical Analysis

Cell nuclei were counted using ImageJ software from FIJI. The statistical analysis was performed using GraphPad Prism 8. One-way ANOVA was used to determine statistical significance between samples.

## 2. Results and Discussion

**Figure 1:**
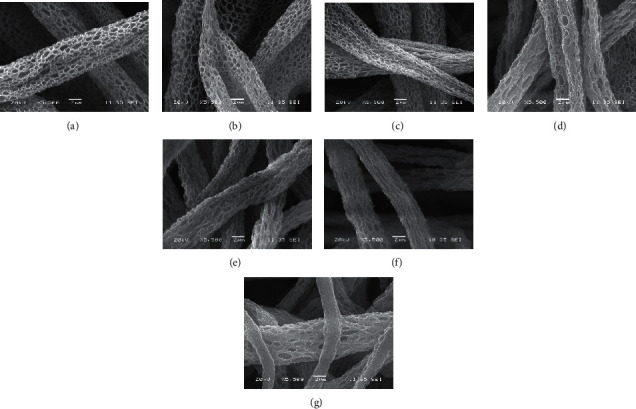
SEM micrograph comparison of the fabricated electrospun mats. (a) CA fiber mat, (b) CA-deacetylated, (c) CA-oxidized, (d) CA-KRSR, (e) CA-BMP-2, (f) CA-RGD, and (g) CA-MP.

SEM was utilized to account for morphological changes in the fiber mats after chemical and biological modifications. The images across the sequential modification of the fiber mats in Figure 1 demonstrate that the fibers retained their surface roughness, and their porosity was not altered. After the biological coupling with all the peptides individually (CA-KRSR, CA-RGD, and CA-BMP-2) and the combination of all (CA-MP), the surface roughness is different although the porous nature of the mats is retained as can be observed (Figures 1(d)–1(g)). Porous morphologies in scaffolds benefit osteointegration since the porous structures in a scaffold are believed to be crucial for cell osteogenesis by allowing mass and fluid transport, cell adhesion, and bone ingrowths [25]. Micropore diameters in macrostructures, such as our fibers, are optimal for osteogenesis as they entrap dissolved Ca^2+^ and PO_4_^3-^ ion concentrations in the pores and, by doing so, decrease the shearing stress present in cells and proteins attached to macropore surfaces [26]. In the natural bone matrix, the coiled and crosslinked microfibrillar structures of collagen type I have a length of ∼300 nm and a diameter of 1.23 nm [15]. In this work, the average diameter of the fibers is maintained between 100 *μ*m and 130 *μ*m and the micropore diameters range from 0.5 um to 1.5 um as can be seen in the histograms in Figure S1.

**Figure 2:**
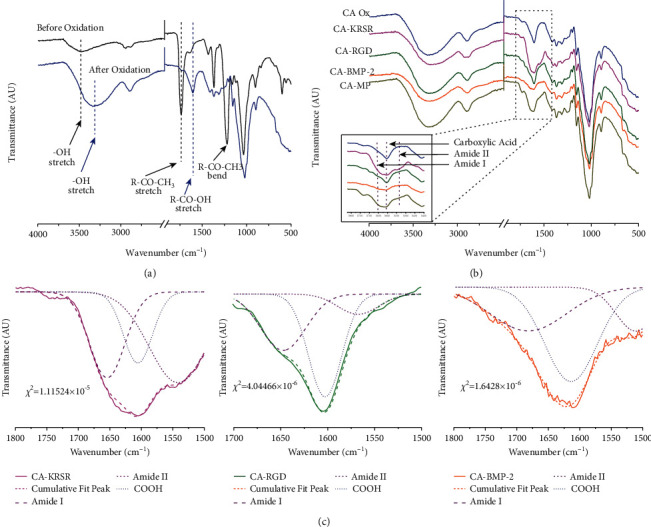
FTIR spectra comparison of the (a) FTIR of CA fiber mat (black) and CA-oxidized (blue) modifications; (b) individual peptide couplings for CA-KRSR (pink), CA-RGD (olive), CA-BMP-2 (orange), and (d) CA-MP (dark yellow); and (c) deconvoluted amide (purple) FTIR peaks using a Gaussian curve fitting function for CA-KRSR (pink), CA-RGD (olive), and CA-BMP-2 (orange).

To confirm the modification of the electrospun fibers, FTIR was conducted to account for characteristic signals (Figures 2(a) and 2(b)). Figure S2 shows the evidence of successful fiber oxidation and modification where a characteristic -OH band in the raw CA fiber mat is present at 3500 cm^−1^. After chemical modifications of the fibers, this band is broadened and shifted to 3320 cm^−1^ and carbonyl group peaks between 1600 and 1750 cm^−1^. This may be due to the increase in H-bonding that occurs upon modifications since the stronger the H-bond, the longer the -OH bond, and thus, the lower the vibrational frequency and the broader and more intense the absorption band. However, the most important information regarding the modifications can be found within the carbonyl peaks. The first carbonyl peak observed belonging to the raw CA occurs at 1750 cm^−1^ due to the ketone (C=O) group of the acetate structure in CA. After deacetylation, this band is lost and, upon oxidation, a new carbonyl band emerges at 1610 cm^−1^ belonging to the carbonyl from the carboxylic acid (C=O). After coupling the peptides (CA-KRSR, CA-RGD, CA-BMP-2, and CA-MP), a change can be observed in the frequency of the characteristic amide carbonyl band around 1,700 cm^−1^ (C=O, amide I) and 1,540 cm^−1^ (N–H amide I). The carbonyl signals belonging to a carboxylic acid group usually occur between 1730 and 1700 cm^−1^; however, these peaks are now shifted to lower frequencies due to the new fiber mat conjugations. In the CA-KRSR sample, the amide band occurs at 1649 cm^−1^ and 1604 cm^−1^, the CA-RGD at 1689 cm^−1^, and the CA-BMP-2 at 1644 cm^−1^. The only peptide-coupled fiber mat with noticeable amide II signal corresponds to the CA-KRSR peptide, which can be attributed to the presence of two arginine amino acids in the structure allowing the vibration of this band. The full untruncated spectra can be seen in Figure S3 where the CA-RGD displays an additional thiol peak corresponding to an impurity as evidenced in Figure S4. As a complimentary technique to corroborate peptide couplings, electron dispersive spectrometry (EDS) was used.  Table S2 shows the elemental composition of each individual peptide-coupled CA fiber mat where the presence of nitrogen can be appreciated. The FTIR amide deconvolution for individual peptide couplings was also performed using a Gaussian curve fitting model with Origin 9 software and is shown in Figure 2(c) [27].

It is known that cellular adhesion to the surface of a biomaterial occurs by (1) attachment, (2) spreading, and (3) focal adhesion to promote a strong interaction, a process mediated by integrins. [19] Given that adhesive proteins attach to the integrin receptors on osteoblast membranes, and the *α*_V_*β*_3_ integrin receptor is one of the primary RGD binding domains, the expression of this receptor was studied. Prior to performing this assay, the presence of the *α*_V_*β*_3_ protein in our cell lines was corroborated using flow cytometry (Figure S5).

**Figure 3:**
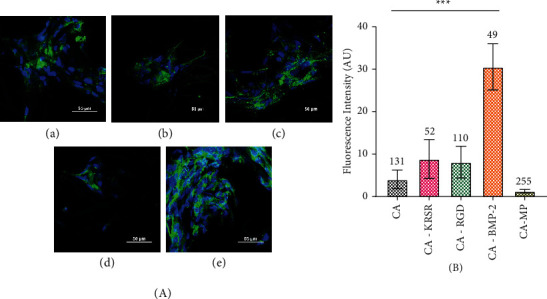
(a) *α*_V_*β*_3_ integrin expression (green) in hFOB 1.19 (blue nuclei) across each CA electrospun mat. (A) CA fiber mat, (B) CA-KRSR, (C) CA-RGD, (D) CA-BMP-2, and (E) CA-MP. Scale bar represents 50 *µ*m. (b) Fluorescence intensity across samples normalized by the total cells displayed over each column.

Qualitatively, the background-reduced CA and CA-MP (Figure 3(a) A and E, respectively) images show a high expression of the integrin receptor. Another observation is that the KRSR peptide (Figure 3(a) B) appears to induce cellular aggregation. The major difference between adhesive RGD and KRSR peptide relies on KRSR having three out of four amino acids positively charged to bind to the negatively charged HS on glycosaminoglycans. Zhang and Webster indicate that there is a strong electrostatic attraction between negatively charged proteins (i.e., fibronectin and vitronectin) and positively charged surfaces. [28] Similar behavior was observed by Chen et al. evaluating osteoblastic actin microfilament arrangement on KRSR-modified surfaces. [29] This implies that the incorporation of positive charges in surfaces can favor preosteoblast adhesion. However, it is also a possibility that the distribution of KRSR peptide throughout the fiber mat was not homogeneous, and cells attached preferably to the KRSR binding sites.

**Scheme 3:**
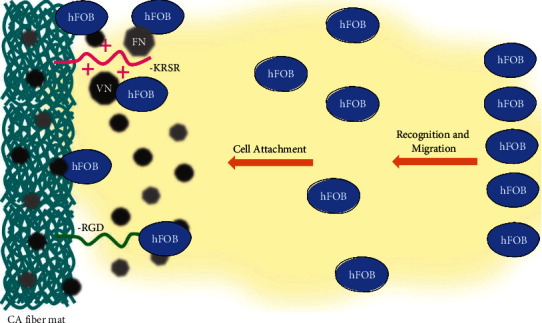
Hypothesized peptide interactions and effect on cellular attachment preference.

Contrary to what was expected, the number of cells present in the CA-RGD (Figure 3(a) C) fiber mat on most images was lower than that in the unmodified CA sample. It is important to distinguish that the adhesive proteins from the culture medium attach to the substrates of biomaterial surfaces in study (in this work, the CA fibers) and cells attach to those adsorbed proteins on the biomaterial surfaces. Hennessy et al. explain that the presence of RGD peptides in combination with adsorbed endogenous proteins might contribute to diminished cell attachment because their presence disrupts the conformations of adsorbed fibronectin (FN) and vitronectin (VN) or because the presence of the RGD peptides blocks the adsorption of endogenous proteins. [30] In this sense, even though RGD peptides have been used to support cell attachment, migration, and differentiation, they reduce binding affinity compared with the same peptide existing as part of its parent protein structure. [31] A representation of these hypothesized interactions is shown in Scheme 3.

The peptides just discussed (KRSR and RGD) were coupled to stimulate cellular adhesion, and similarly, the fiber mat treated with BMP-2 peptide growth factor showed lesser cells against other samples (Figure 3(a) D). It is known that BMPs have significant effects on human osteoblast cells between 10 and 1000 pg/ml in vitro. [32] However, it is important to note that the effect of the concentration of natural BMP-2 is not necessarily equal, to the effect of peptide concentration. And, since the real concentration of peptide present in the fiber mats is unknown, the low number of cells could be due to the concentration of the peptide in the fiber mat being too high inducing a contrary and toxic response to the cells. Saito et al. studied the activation of osteo-progenitor cells by the BMP-2 synthetic peptide corresponding to the knuckle epitope of BMP-2, the same sequence used in this study, and found that when cocultured alongside parent rh-BMP-2 protein, the fluorescence exhibited by the cells with the peptide alone disappeared. [33] This could mean that the absence of the synergy domains of BMP-2 in the peptide leads to poor osteoconduction. In our study, after comparing fluorescence intensity (Figure 3(b)), the CA-BMP-2 sample was the only significant value against untreated CA fiber mat and showed the highest fluorescence intensity signal while the number of nuclei (49) was lesser than in the other fiber mats. Likewise, the CA-MP fiber mat had the highest number of cells (255), whereas its fluorescence intensity was the lowest.

Integrins, like *α*_V_*β*_3_, are constantly switching between inactive bent and active extended conformations responsible for adhesion initiation after ligand binding [34]. The crucial step in integrin activation is considered to be the binding of intracellular proteins, such as talins and kindlins, to the tail of *β*-integrins that cluster into focal complexes for attachment while inactive integrins are usually found disordered across the cell or dorsally [35]. Overall, integrin activation involves a series of steps that include conformational changes, engagement with extracellular ligands, connections with the actin cytoskeleton, clustering to form focal adhesions, and signal transductions, all leading to integrin inactivation, which occurs by Ser/Thr phosphorylation of the talin and kindlin binding motif (NPXY) [36, 37]. Inactive integrins diffuse rapidly in the plane of the membrane because focal adhesions disassemble to recycle integrins and permit new adhesion sites [38]. By analyzing the distribution and quantity of *α*_V_*β*_3_ integrins present, we can suggest that the fluorescence intensity is indirectly proportional to the integrin activity and thus the fiber mat treatment, which activated *α*_V_*β*_3_ to a larger degree corresponds to CA-MP followed by untreated CA. Additionally, it is possible that the incorporation of the BMP-2 peptide does not activate the *α*_V_*β*_3_ integrin considering that the purpose of its incorporation was not for adhesion but for later maturation. A concluding remark with certainty towards integrin activation evaluation cannot be offered with this data. A more accurate method to determine such information would be to use an active/inactive antibody that is specific for this integrin or a variation in *α*_V_*β*_3_ secondary antibody that could be used with a phosphorylation tag.

It is important to also acknowledge that the definite concentration of the peptides present in the CA fiber mats as an unknown variable and that it is also possible that there was a competition between all three peptides to couple to the fiber mats in the CA-MP sample, which could explain why qualitatively there were more cells found in this fiber mat. Yet, to attribute the increased expression observed in CA-MP to either one of the peptides would be incorrect. As previously discussed for KRSR and RGD peptides, another factor that needs to be acknowledged is that there may exist competition between the BMP-2 peptide and the actual BMP-2 protein present. The results from the control plates (Figure S6) showed that there were fewer cells present in the fiber mats to which it can be concluded that at short incubation periods, the addition of peptides to the culture medium decreases the affinity of the cells to attach to the fibers.

### 2.1. Cell Morphology

**Figure 4:**
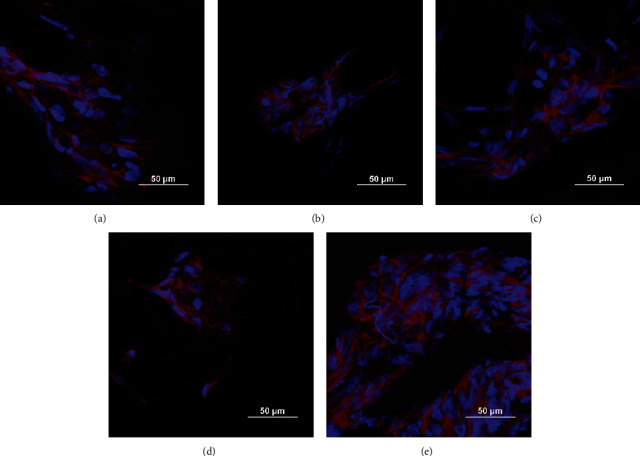
Cytoskeleton (red) of the hFOB 1.19 (blue nuclei) at 7 days of culture in each CA electrospun mat. (a) CA fiber mat, (b) CA-KRSR, (c) CA-RGD, (d) CA-BMP-2, and (e) CA-MP. Scale bar represents 50 *µ*m.

Osteoblastic cell lines are adherent cell types. It is important for biomaterials to provide a favorable environment for cells to attach and expand by developing filopodia associated with cell migration and communication. The addition of Phalloidin 647 iFluor reagent was carried out for the purpose of marking the cellular actin filaments composing the cell's cytoskeleton. The morphology of the cells was studied across all assays, but only present the Phalloidin staining at 7 days due to increased and overlapping cells at 14 days. The morphology of the cells in the fiber mats (Figure 4) indicates that there is no effect of the peptide presence or absence against the cellular cytoskeleton. The same extended shape that is seen in the untreated fiber mat is repeated throughout all samples. In a 2D culture system (i.e., glass discs), the morphology of the osteoblastic lines appears as elongated and flat linear shapes, but the morphology is interpreted differently in a 3D system such as this one. The elongation of the cells is dependent on the fibrous arrangement in which the cell is adhered. Since the fibers in the mats are not oriented in a specific direction, the shape of the cells will surround the shape of the fibers.

### 2.2. Cell Proliferation

**Figure 5:**
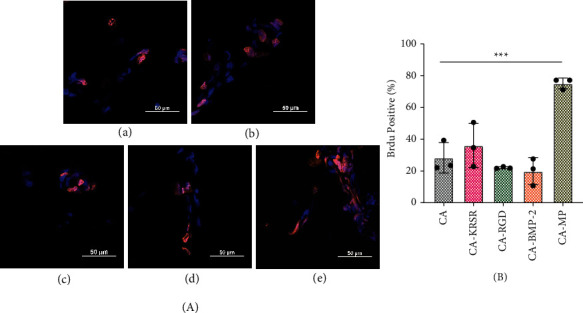
(a) hFOB 1.19 BrdU cell proliferation assay against each CA electrospun mat. Blue nuclei represent total cells, and orange overlap represents the BrdU-incorporated nuclei in (A) CA fiber mat, (B) CA-KRSR, (C) CA-RGD, (D) CA-BMP-2, and (E) CA-MP. Scale bar represents 50 *µ*m. (b) BrdU-positive cells in each treated fiber mat.

BrdU is a thymidine analogue that is incorporated into the cell DNA at the S phase of cell cycle and allows the determination and quantification of cellular reproduction.  Figure 5(a) presents some images of the BrdU assay where the total number of nuclei present can be seen by the blue DAPI staining including the BrdU-incorporated nuclei seen with orange overlaps. The total number of BrdU-positive cells was adjusted by the total number of cells present in each sample and expressed as percentages in Figure 5(b). The values represent mean ± standard deviation (SD) from three replicates. Triple asterisk indicates statistical significance against untreated CA fiber mat sample (*p*<0.0002). The total amount of cells that attached to the fiber mats is shown in Figure S7 where it is suggested that the low number is due to the following reasons: (1) the cultured cell concentration was too low to be significant in (2) a 3-dimensional scaffold where the focusing could miss other nuclei present at a different focal lengths as per described in the acquisition methodology used. Examining cell proliferation by itself in a period of hours is not a mere indicator of the bioactivity of a material unless complimented with other assays. Other possible explanations that justify lower than expected cell numbers and proliferation were discussed already. Regardless of the cell numbers in this assay, the greatest proliferation rate was found for CA-MP, which is in accordance with the elevated number of cells found for this treatment (255) in the previous adhesion assay analysis as well

### 2.3. hFOB Differentiation and ECM Formation on Mats

To evaluate cellular development, the cell culture incubation time was extended to a period of 14 days to study the expression of maturation markers such as collagen and bone sialoprotein (BSP) as these are secreted from the cells when they are in the process of forming the bone matrix.

#### 2.3.1. Collagen

**Figure 6:**
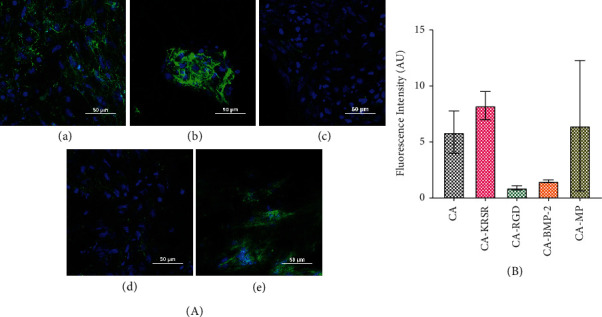
(a) Collagen I (green) assay for hFOB 1.19 (blue nuclei) across each CA electrospun mat. (A) CA Fiber mat, (B) CA-KRSR, (C) CA-RGD, (D) CA-BMP-2, and (E) CA-MP. Scale bar represents 50 *µ*m. (b) Alexa Fluor 488 Fluorescence Intensity plot normalized by the number of cells for collagen expression. Error bars represent the standard error of the mean.

Collagen is an indicator of osteoblast differentiation as these cells secrete large amounts of type I collagen (with small quantities of matrix organizing proteins that include osteocalcin and osteopontin) [39]. Initially, bone collagen is secreted as a propeptide, and after enzymatic cleavage, collagen molecules align and form functional fibrils. The results shown in Figure 6(a) display the abundance of collagen fibers according to representative images from quantitative data shown after. The CA-KRSR sample displayed cellular aggregation just like in the results seen in Figure 6(a) B, yet the control plate (Figure S9) showed a larger network of collagen evenly distributed across the sample. This could be due to the previously discussed reasoning implying that native proteins attach to the fibers while the peptides are continuously washed away with each medium replacement, and specifically, the KRSR peptide has a positive charge to which we could attribute the preference of serum proteins to attach to the CA fibers over the peptides. Figure 6(b) shows quantitative results obtained from fluorescence intensity values and normalized by the total number of cells found in the images used for obtaining intensity values. There was no significant difference found between any of the treatments. In this experiment, the conjugation of the peptides is not promoting long-term effects over differentiation and maturation. Figure S11 shows the Phalloidin-stained cytoskeleton of the cells in this assay.

#### 2.3.2. Bone Sialoprotein (BSP)

**Figure 7:**
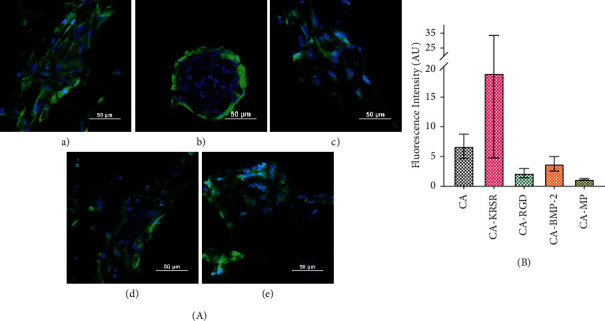
(a) Bone sialoprotein (green) assay for hFOB 1.19 (blue nuclei) across each CA electrospun mats. (A) CA Fiber mat, (B) CA-KRSR, (C) CA-RGD, (D) CA-BMP-2, and (E) CA-MP. Scale bar represents 50 *µ*m. (b) Alexa Fluor 488 Fluorescence Intensity plot for BSP expression normalized by the total cells. Error bars represent the standard error of the mean.

Osteoblast differentiation can also be assessed by the deposition of Ca^2+^ and PO_4_^3−^ minerals who form the mineral matrix of hydroxyapatite. Bone sialoprotein (BSP) binds tightly to hydroxyapatite crystals, which are deposited in the gaps between collagen fibers. This assay provides important information about the cell-matrix interaction. The results in Figure 7(a) show evidence of BSP expression adjusted relative to quantitative fluorescence intensity data throughout all the fiber mats. The BSP expression is homogeneous across the fibers in all the samples, and thus, the fibers provide good surface morphology for osteoblastic cell proliferation and maturation. Although not as notable as in Figure 7(a), the control samples with the peptides in solution (Figure S10) also demonstrated evidence of BSP expression. The CA-KRSR fiber mat (Figure 7(a)B) continues showing cellular aggregation, yet the cells continue to grow and form a new matrix. Reasons for this behavior were discussed previously. Similar to the previous collagen assay, the quantitative data representing the fluorescence intensity plot normalized by the total cells found in the images used for obtaining intensity values did not show significant differences across either fiber mat treatment, sustaining the previous statement about long-term effects of peptide couplings. Figure S12 shows the Phalloidin-stained cytoskeleton of the cells in this assay.

**Figure 8:**
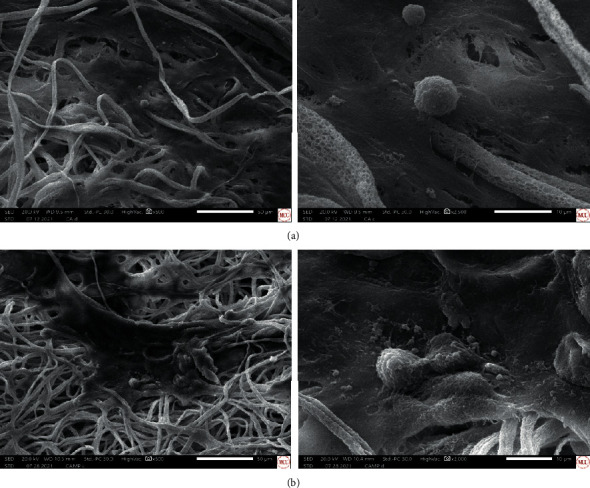
SEM images of hFOB 1.19 cells on (a) CA and (b) CA-MP fiber mats after 14 days of incubation.

As known, the maturation of the cells leads to collagen formation where hydroxyapatite crystals are deposited. The secretion of calcium deposits was assessed with electron dispersive spectroscopy (EDS) after a 14-day incubation period with the CA and CA-MP fiber mats.  Figure 8 shows the evidence of anchoring activity from the cells to the fiber mats through the presence of extended filopodia. The images also show the presence of spheric protuberances, which are indicative of mineralization confirmed by the presence of calcium and phosphorous by EDS (Figure S13).

## 3. Conclusion

This manuscript presents the fabrication of a porous fiber mat obtained by the electrospinning technique. The mats were chemically modified to conjugate adhesive- and growth-promoting peptides to render the fiber mat bioactive. Upon bioactivation, the physical properties did not change, and the porous surface morphology provided rough surfaces that allowed preosteoblastic cells to attach and grow. No specific individual peptide prompted contrasting biological responses. At the short culture time of 48 h, all individual peptides appeared to reduce the number of cells present compared to the unmodified CA fiber mat which, as explained, can be due to the peptides blocking the adsorption of adhesive proteins. Yet, when extending to a longer culture period, to evaluate the expression of adhesive protein *α*_v_*β*_3_, there was a statistically significant difference in both the cell numbers and the expression of this protein appearing to have an inverse relationship. The CA-MP fiber mat displayed a much larger number of cells present to which we can conclude that the peptides synergistically had a positive effect on cellular adhesion to the fiber mats. Afterwards, at 14 days of culture, all cells were able to differentiate into bone building cells having the capability to synthesize ECM proteins such as type I collagen and BSP although neither treatment resulted in significant differences between groups. In terms of the capacity of cells to migrate across a surface, all cells were able to attach to the fibers in their usual extended manner exhibiting their characteristic actin fiber extension and in SEM images filopodia protrusions in the cell membrane. Peptides in culture media decreased the affinity of the cells to attach to the fibers; therefore, the inclusion of peptides in the development of biomaterials could prove to be more beneficial using a covalent strategy for initial attachment. The correlation between the amount of peptide present and the expression of the proteins remains; nevertheless, it can be concluded that the peptide coupling approach proves to be a potential alternative to further explore and implement in the fields of bone tissue engineering (BTE). In this study, we presented the coupling of several peptides, each having a different function, and found that their presence together (CA-MP) induced greater proliferation, increased integrin activation, but had no significant effect for long-term maturation. Therefore, the inclusion of adhesive- and growth-promoting peptides into biomaterials to promote early osteoconduction can become a foundation for future scaffold development strategies that result in the regeneration of bone tissue.

## Data Availability

Data are included within the supplementary information.

## Supplementary Materials

Supplementary Materials Supplementary information includes the following tables and figures. Table S1: bioactive peptide sequences, Figure S1: FTIR spectra of CA fiber mat chemical modifications in preparation for peptide conjugations. Figure S2: histograms representing the size distribution of the CA fibers in the CA mats across each modification and peptide addition. Figure S3: Untruncated FTIR spectra of peptide conjugations. Figure S4: GenScript provided Certificate of Purity for RGD peptide displaying HPLC analysis. Table S2: elemental composition results of bioactive CA fiber mats by EDS. Figure S5: flow cytometry of hFOB 1.19 (a) population chosen, (b) control fluorescence, (c) antibody fluorescence, (d) untreated control, (e) secondary only control and (f) antibody-treated cells. Scheme S1: well plate design for the controls used in the ICC assays including (a) untreated glass disc positive control (with primary and secondary corresponding antibodies), (b) untreated glass disc negative control (with only the secondary corresponding antibody), CA fiber mat with corresponding peptides (c) KRSR, (d) RGD, (e) BMP-2, and (f) all peptides in solution. Figure S6: BrdU cell proliferation assay for hFOB 1.19. Blue nuclei represent total cells, and orange overlap represents the BrdU-incorporated nuclei in (a) positive disc control, (b) CA + KRSR, (c) CA + RGD, (d) CA + BMP-2, and (e) CA + MP in DMEM. Figure S7: total cells attached to each fiber mat. This total was used to normalize BrdU-positive cells and express them as a percentage on Figure 5 B. Figure S8: hFOB 1.19 Expression of *α*V*β*3 integrin (green) on (a) positive disc control, (b) CA + KRSR, (c) CA + RGD, (d) CA + BMP-2, and (e) CA + MP in DMEM. Figure S9: hFOB 1.19 Expression of collagen I (green) in (a) positive disc control, (b) CA + KRSR, (c) CA + RGD, (d) CA + BMP-2, and (e) CA + MP in DMEM. Figure S10: hFOB 1.19 expression of BSP (green) in (a) positive disc control, (b) CA + KRSR, (c) CA + RGD, (d) CA + BMP-2, and (e) CA + MP in DMEM. Figure S11: Phalloidin-stained hFOB 1.19 cytoskeleton (red) at 14 days from collagen assay. Figure S12: Phalloidin-stained hFOB 1.19 cytoskeleton (red) at 14 days from BSP assay. Figure S13: EDS of hFOB 1.19 seeded on (a) CA and (b) CA-MP fiber mats.
