# Supplementary material for: Bioactive Cellulose Acetate Electrospun Mats as Scaffolds for Bone Tissue Regeneration
Source: Int J Biomater. 2022 Feb 4;2022:3255039. doi: 10.1155/2022/3255039 (PMC8837436; doi:10.1155/2022/3255039)
Supplement: Supplementary Materials — Supplementary information includes the following tables and figures. Table S1: bioactive peptide sequences, Figure S1: FTIR spectra of CA fiber mat chemical modifications in preparation for peptide conjugations. Figure S2: histograms representing the size distribution of the CA fibers in the CA mats across each modification and peptide addition. Figure S3: Untruncated FTIR spectra of peptide conjugations. Figure S4: GenScript provided Certificate of Purity for RGD peptide displaying HPLC analysis. Table S2: elemental composition results of bioactive CA fiber mats by EDS. Figure S5: flow cytometry of hFOB 1.19 (a) population chosen, (b) control fluorescence, (c) antibody fluorescence, (d) untreated control, (e) secondary only control and (f) antibody-treated cells. Scheme S1: well plate design for the controls used in the ICC assays including (a) untreated glass disc positive control (with primary and secondary corresponding antibodies), (b) untreated glass disc negative control (with only the secondary corresponding antibody), CA fiber mat with corresponding peptides (c) KRSR, (d) RGD, (e) BMP-2, and (f) all peptides in solution. Figure S6: BrdU cell proliferation assay for hFOB 1.19. Blue nuclei represent total cells, and orange overlap represents the BrdU-incorporated nuclei in (a) positive disc control, (b) CA + KRSR, (c) CA + RGD, (d) CA + BMP-2, and (e) CA + MP in DMEM. Figure S7: total cells attached to each fiber mat. This total was used to normalize BrdU-positive cells and express them as a percentage on Figure 5 B. Figure S8: hFOB 1.19 Expression of αVβ3 integrin (green) on (a) positive disc control, (b) CA + KRSR, (c) CA + RGD, (d) CA + BMP-2, and (e) CA + MP in DMEM. Figure S9: hFOB 1.19 Expression of collagen I (green) in (a) positive disc control, (b) CA + KRSR, (c) CA + RGD, (d) CA + BMP-2, and (e) CA + MP in DMEM. Figure S10: hFOB 1.19 expression of BSP (green) in (a) positive disc control, (b) CA + KRSR, (c) CA + RGD, (d) CA + BMP-2, and (e) CA + MP [file 3255039.f1.docx]

| **Table S1: Bioactive Peptide Sequences** | | |
| --- | --- | --- |
| Abbreviation | Structure | MW (g) |
| KRSR | (Ahx)(Ahx)(Ahx)KRSR  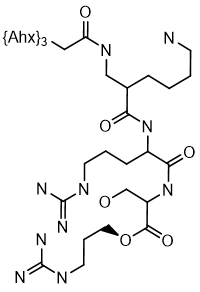 | 885.2 |
| RGD | (Ahx)(Ahx)(Ahx)GRGD  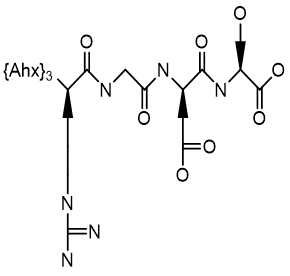 | 830.0 |
| BMP-2 | (Ahx)(Ahx)(Ahx)KIPKASSVPTELSAISTLYL  | 2457.6 |





**Figure S1**: Histograms representing the size distribution of the CA fibers in the CA Mats across each modification and peptide addition





**Figure S2**: FTIR spectra of CA Fiber Mat chemical modifications in preparation for peptide conjugations





**Figure S3**: Untruncated FTIR spectra of peptide conjugations

The full untruncated FTIR spectra of the RGD coupled CA fiber mat shows a weak band occurring at 2338 cm^-1^ which corresponds to the region of a thiol (-SH) group. None of the peptides exhibit such group and this thiol may correspond to an impurity present from the peptide synthesis which matches the certificate of purity during manufacturing where the HPLC curve displayed 3 additional peaks.


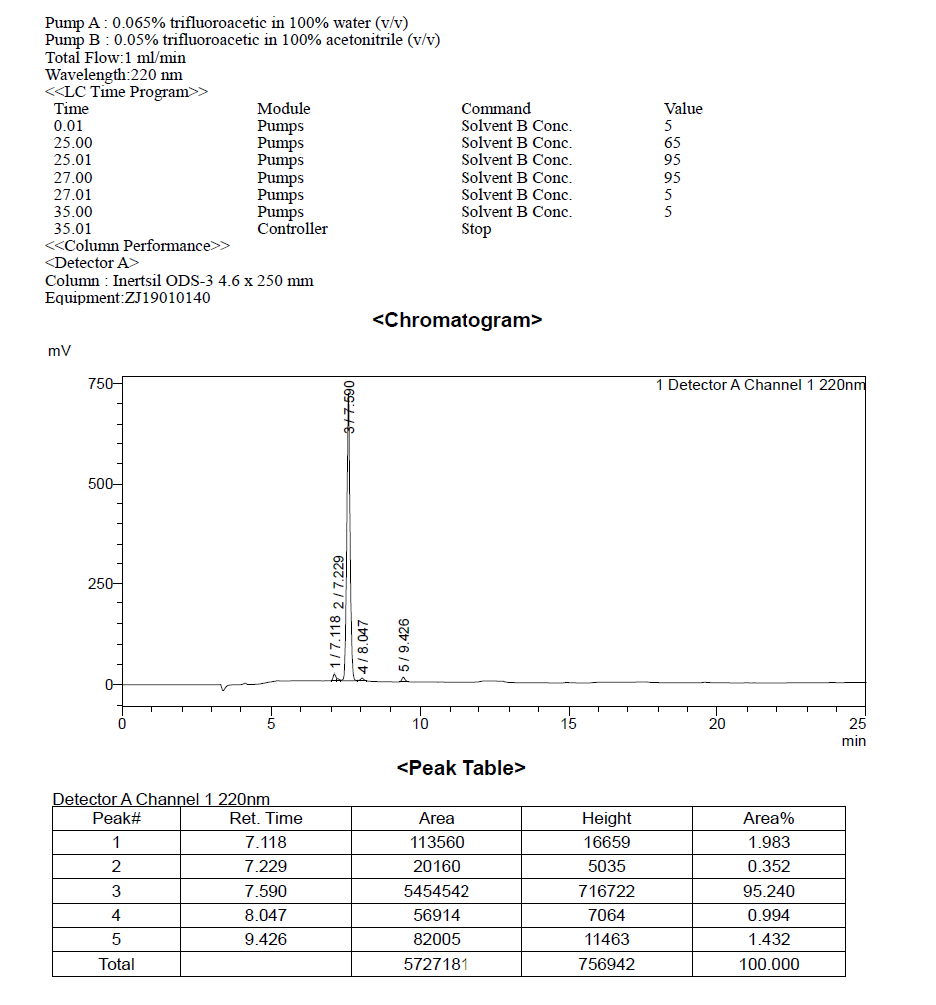


**Figure S4**: Genscript provided Certificate of Purity for -RGD peptide displaying HPLC analysis

| **Table S2 Elemental Composition Results of Bioactive CA Fiber Mats by EDS** | | |
| --- | --- | --- |
| CA-KRSR | CA-RGD | CA-BMP-2 |
|  | 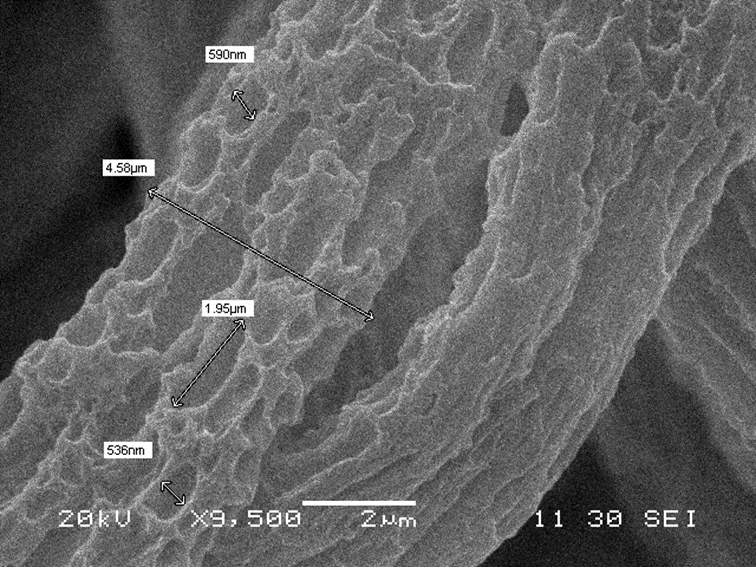 | 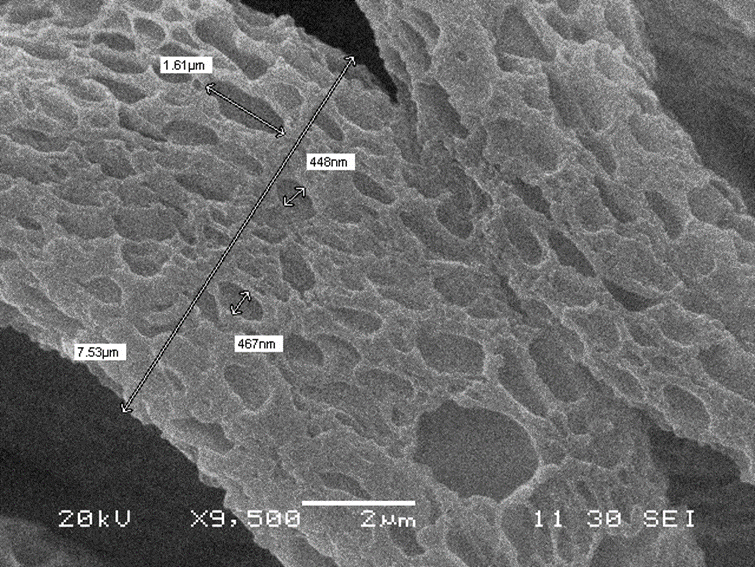 |
| 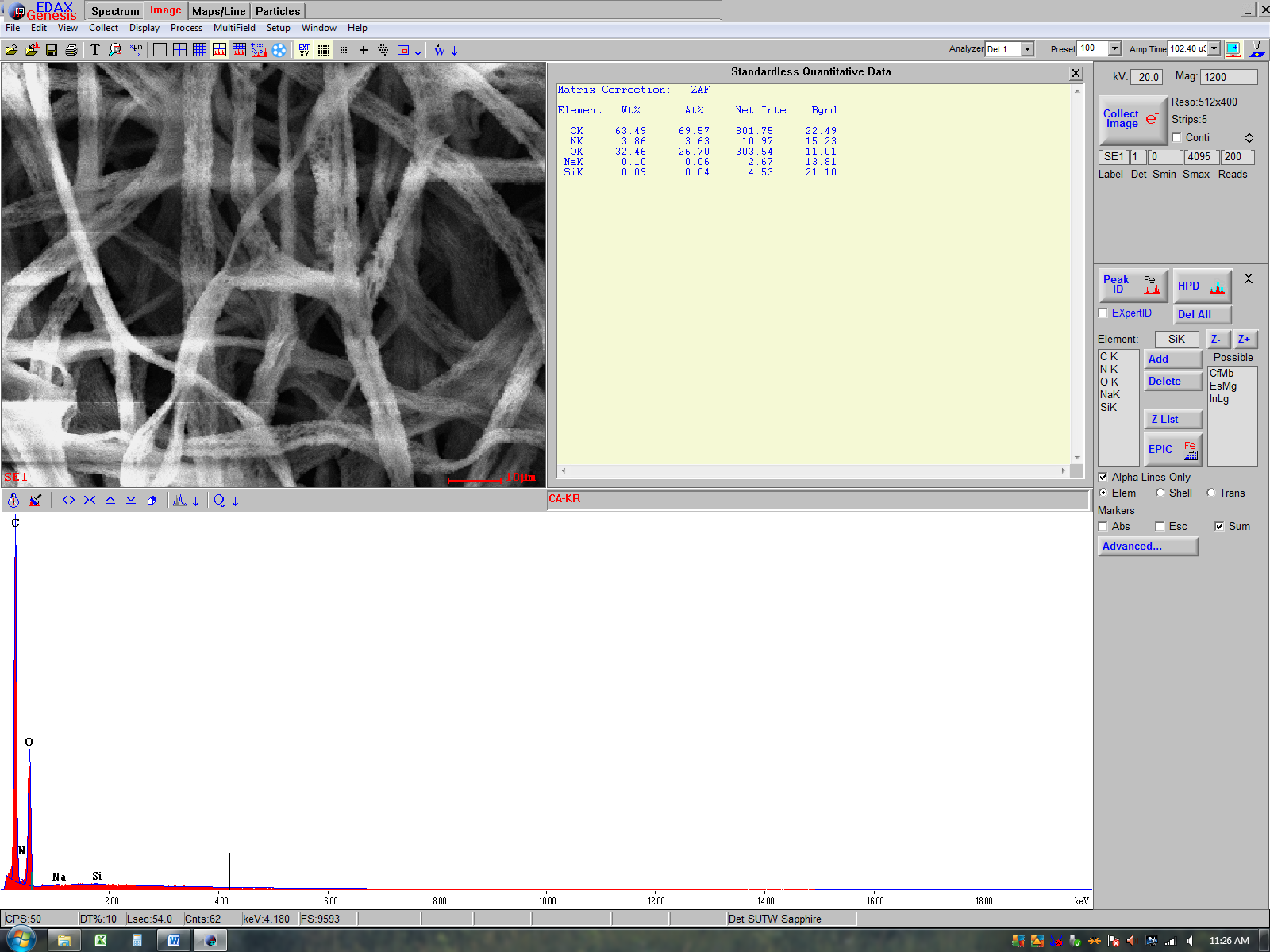 | 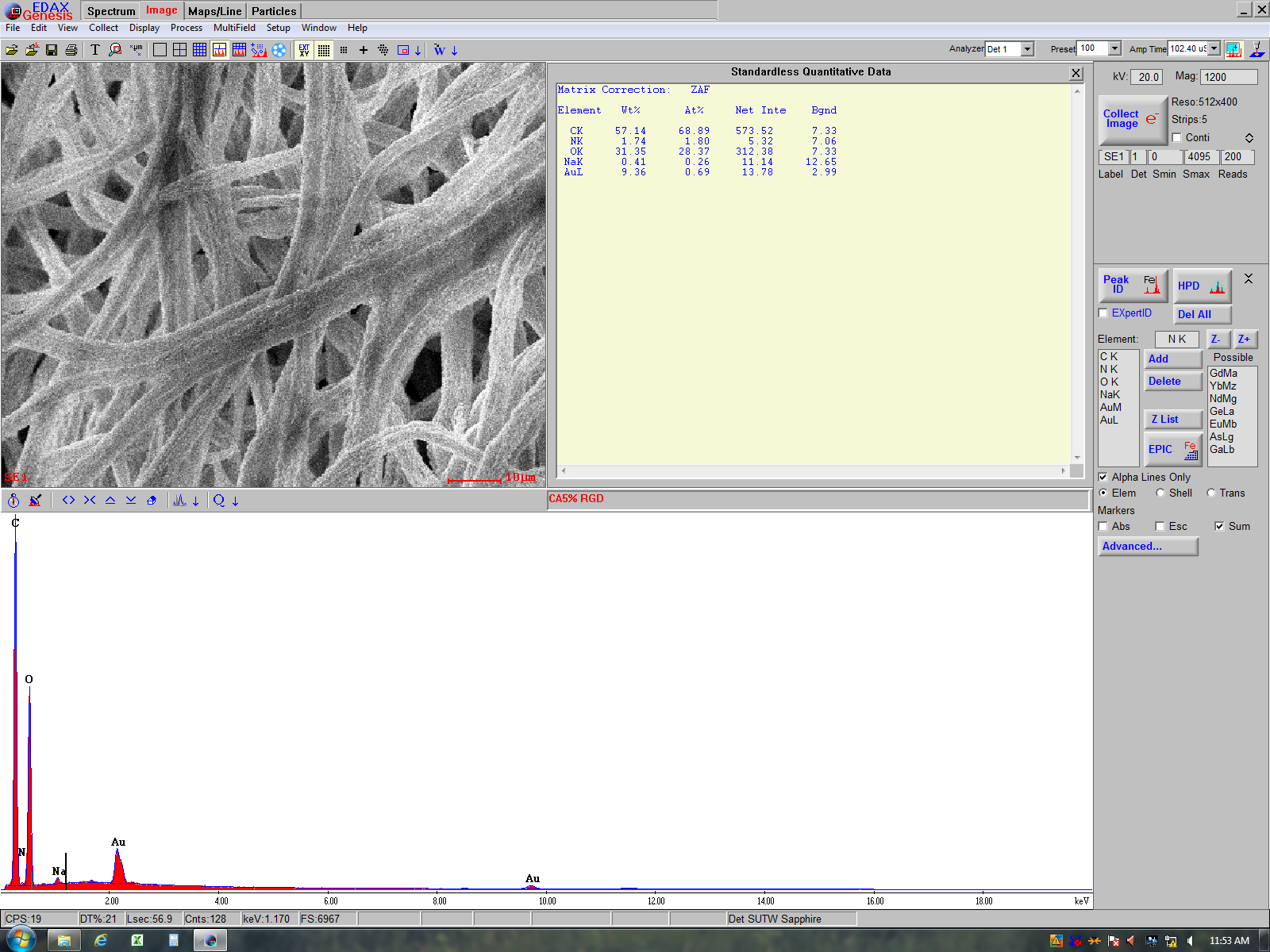 | 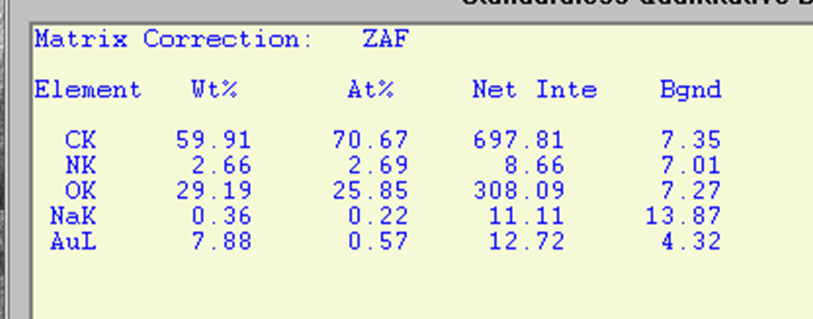 |


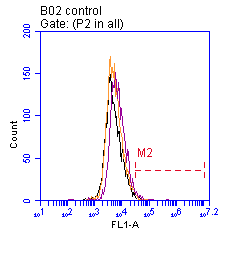


b)


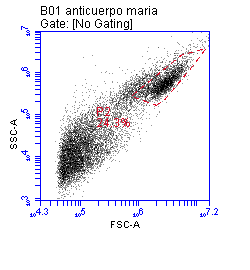


a)


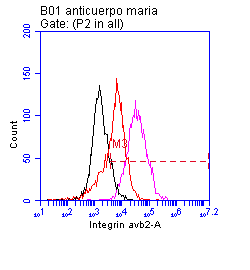


c)


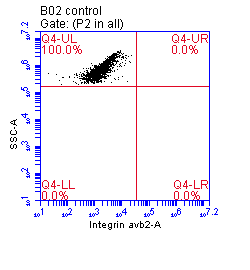


d)


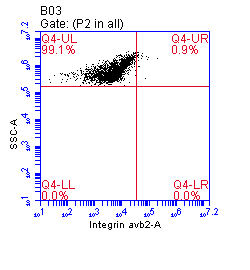


e)


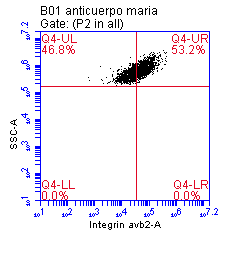


f)

**Figure S5:** Flow Cytommetry of hFOB 1.19 a) Population Chosen, b) Control Fluorescence, c) Antibody Fluorescence, d) Untreated Control, e) Secondary Only Control and f) Antibody Treated Cells


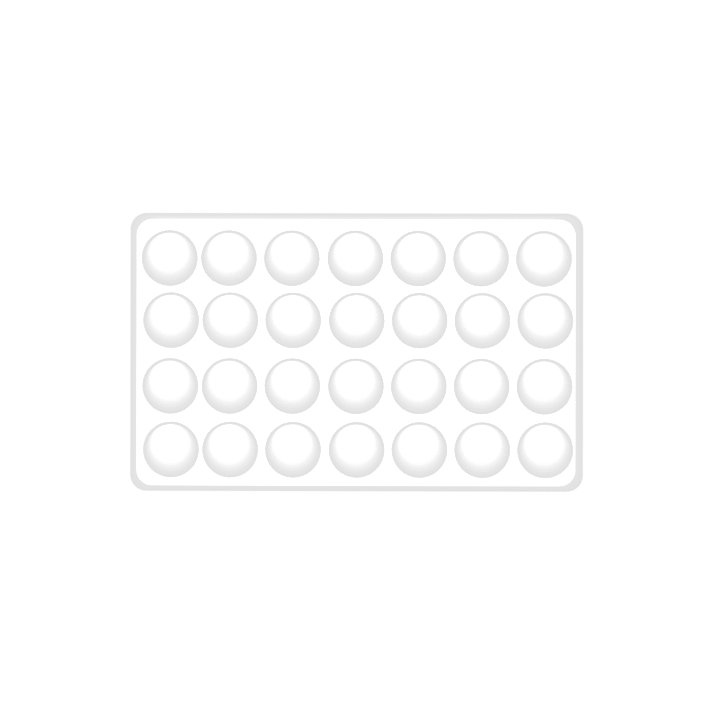


C) DMEM + KRSR

D) DMEM + RGD

E) DMEM + BMP-2

F) DMEM + KRSR +RGD + BMP-2

A

B

**Scheme S1**: Well plate design for the controls used in the ICC assays including a) Untreated Glass Disc Positive Control (with primary and secondary corresponding antibodies), b) Untreated Glass Disc Negative Control (with only the secondary corresponding antibody), CA Fiber Mat with corresponding peptides c) KRSR, d) RGD, e) BMP-2 and f) All peptides in solution

| 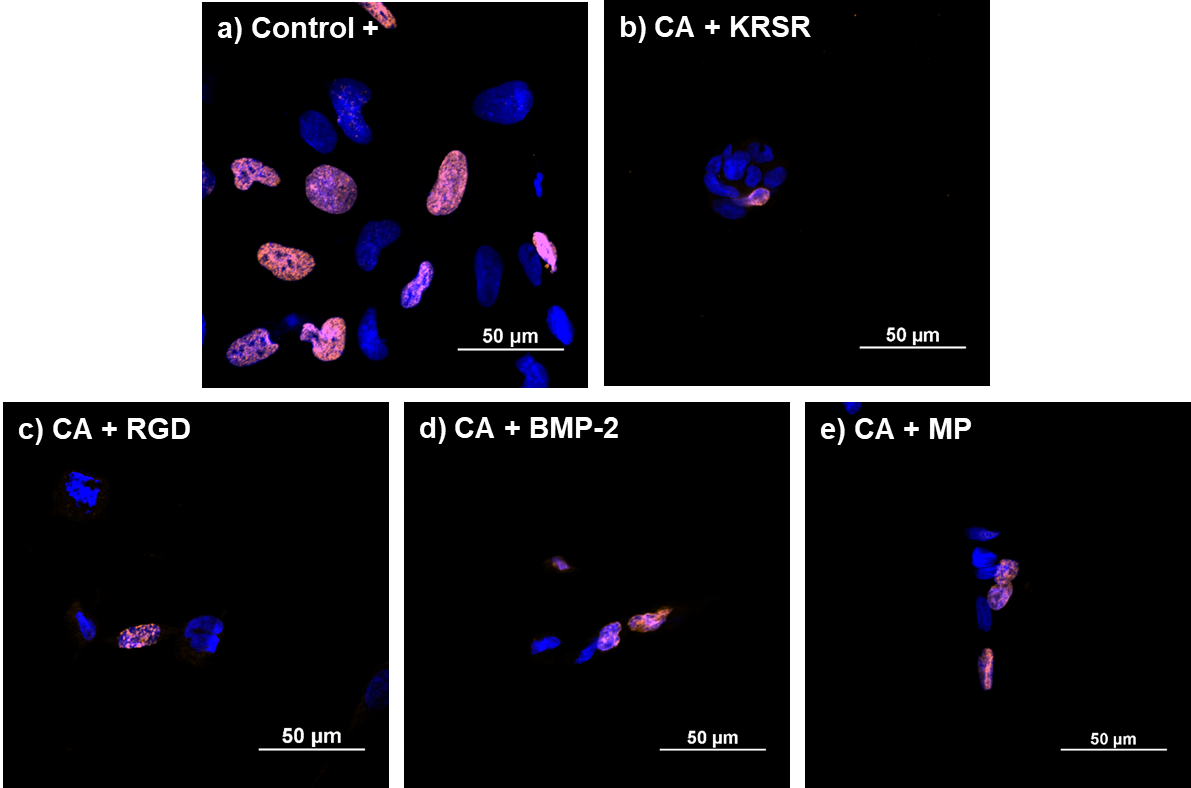 |
| --- |
| **Figure S6** BrdU cell proliferation assay for hFOB 1.19. Blue nuclei represent total cells and orange overlaps represents the BrdU incorporated nuclei in a) Positive Disc Control, b) CA + KRSR, c) CA + RGD, d) CA + BMP-2 and e) CA + MP in DMEM. Scale bar represents 50 µm. |





**Figure S7** Total cells attached to each Fiber Mat. This total was used to normalize BrdU positive cells and express them as a percentage on Figure 5 B.

| 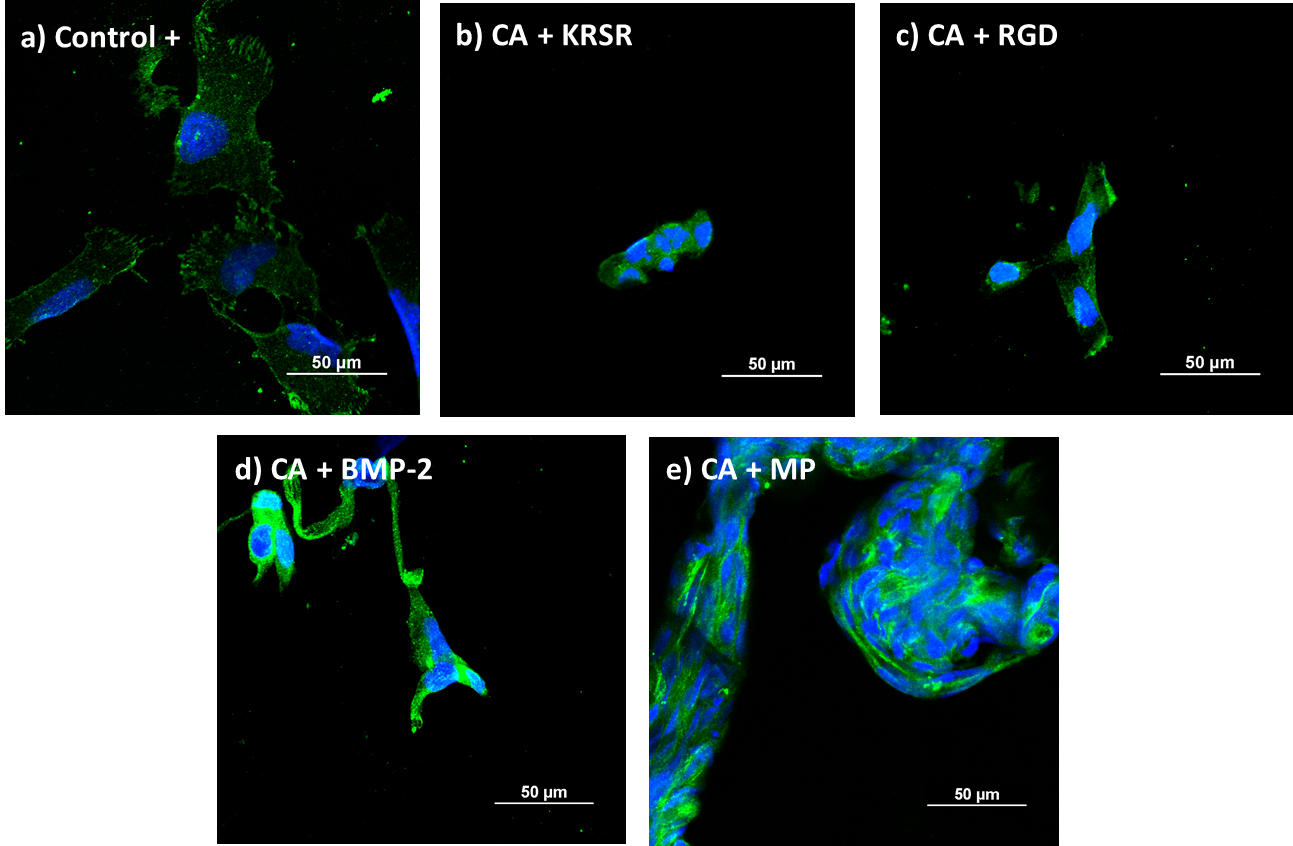 |
| --- |
| **Figure S8:** hFOB 1.19 Expression of α_V_β_3_ integrin (green) on a) Positive Disc Control, b) CA + KRSR, c) CA + RGD, d) CA + BMP-2 and e) CA + MP in DMEM. Scale bar represents 50 µm. |

| 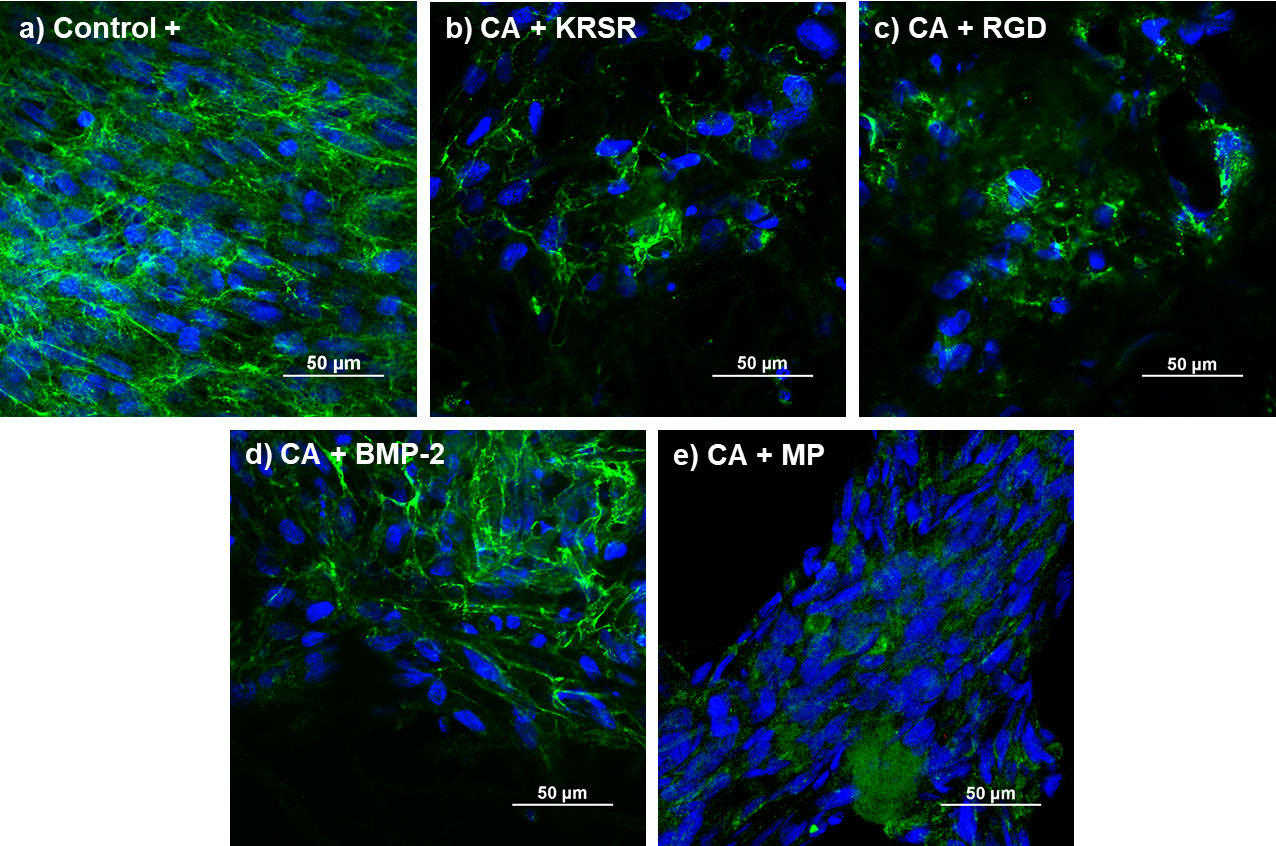 |
| --- |
| **Figure S9**: hFOB 1.19 Expression of Collagen I (green) in a) Positive Disc Control, b) CA + KRSR, c) CA + RGD, d) CA + BMP-2 and e) CA + MP in DMEM. Scale bar represents 50 µm. |

| 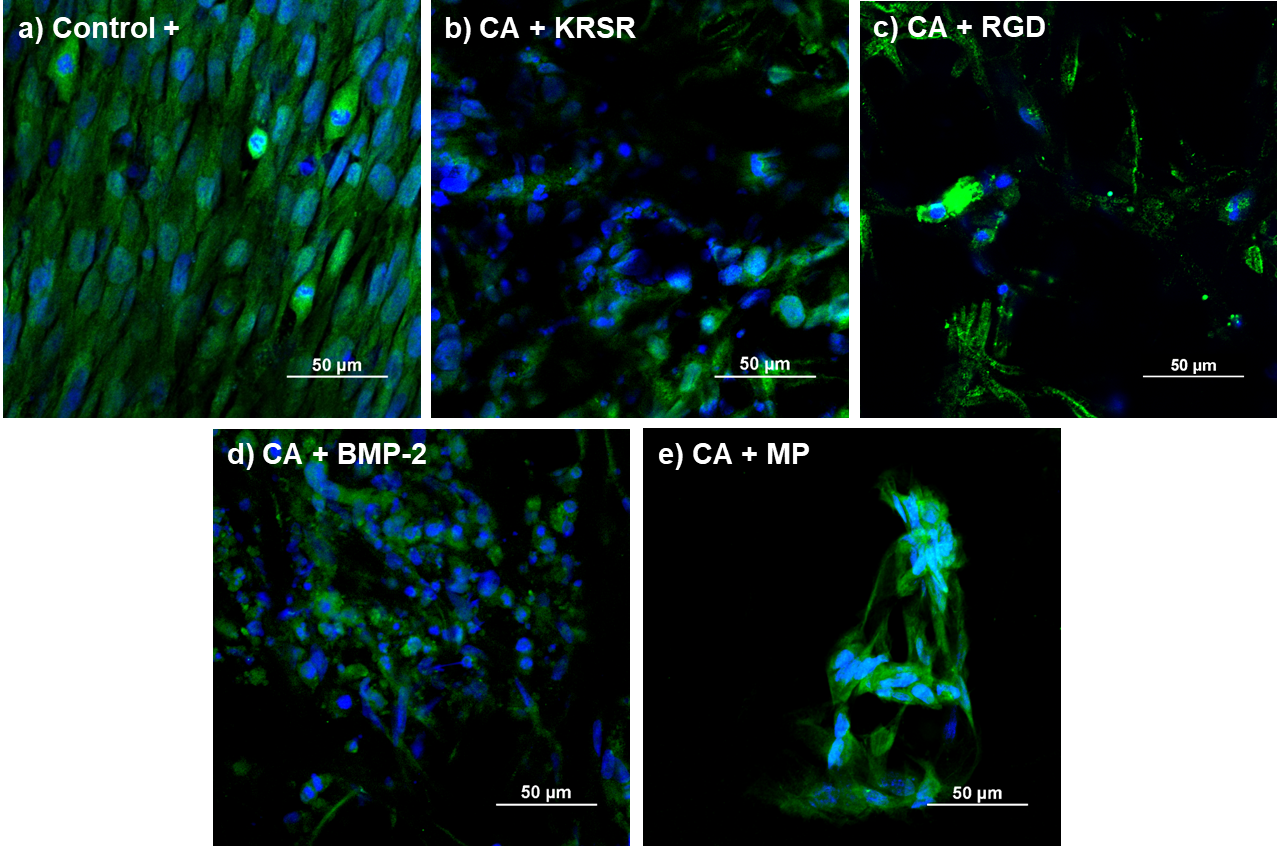 |
| --- |
| **Figure S10**: hFOB 1.19 Expression of BSP (green) in a) Positive Disc Control, b) CA + KRSR, c) CA + RGD, d) CA + BMP-2 and e) CA + MP in DMEM. Scale bar represents 50 µm. |

| 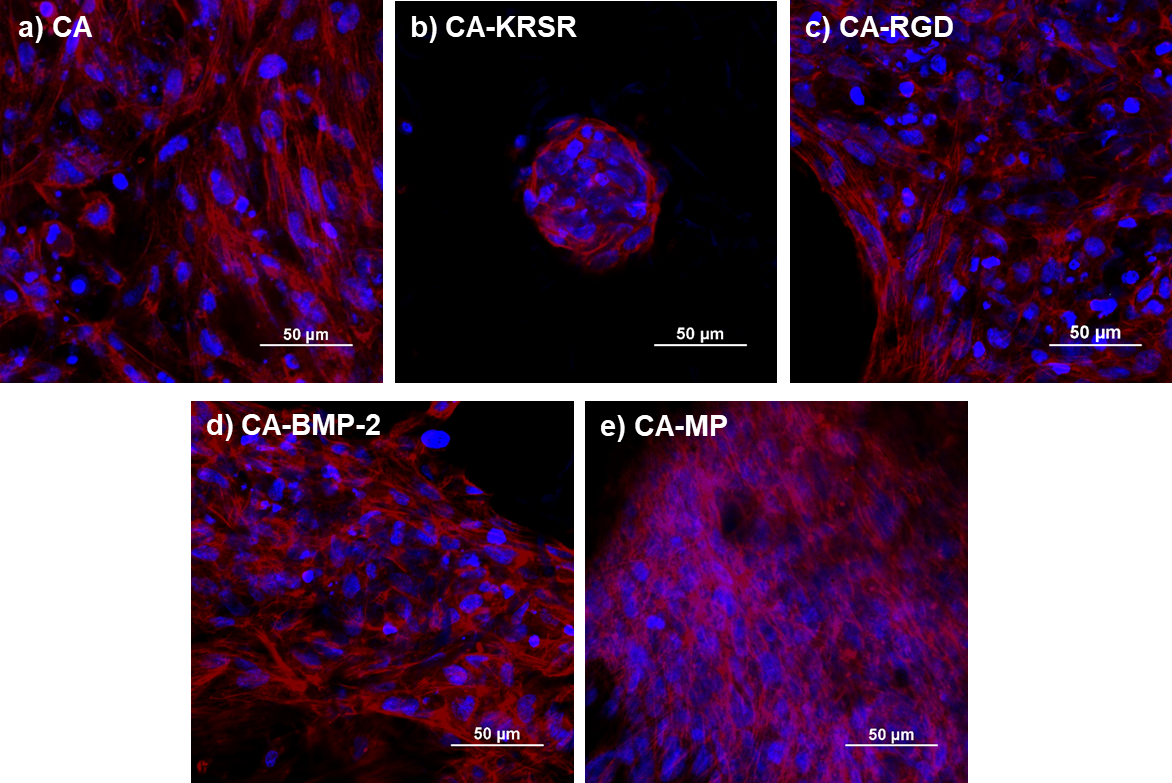 |
| --- |
| **Figure S11**: Phalloidin Stained hFOB 1.19 Cytoskeleton at 14 days from Collagen Assay. Scale Bar Represents 50 µm. |

| 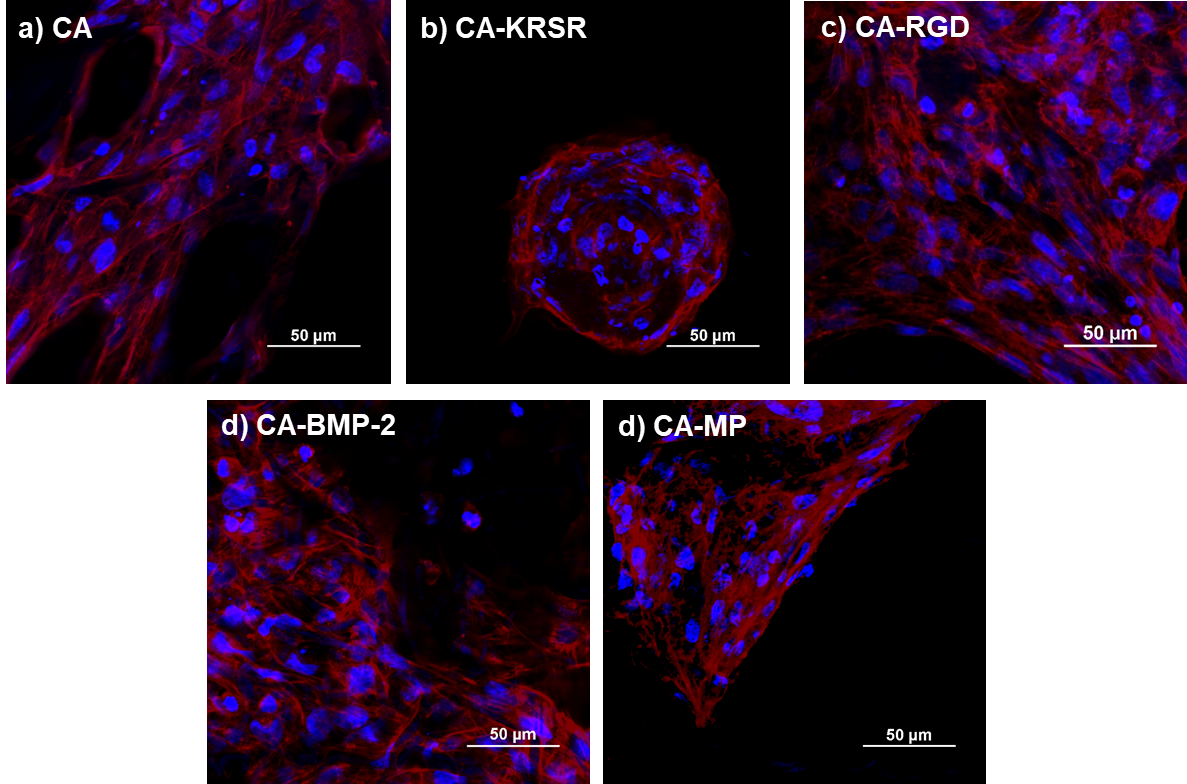 |
| --- |
| **Figure S12**: Phalloidin Stained hFOB 1.19 Cytoskeleton at 14 days from BSP Assay. Scale Bar Represents 50 µm. |

| a)  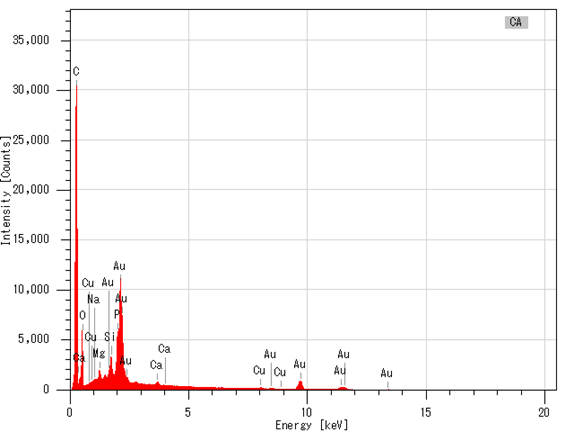 |
| --- |
| b)  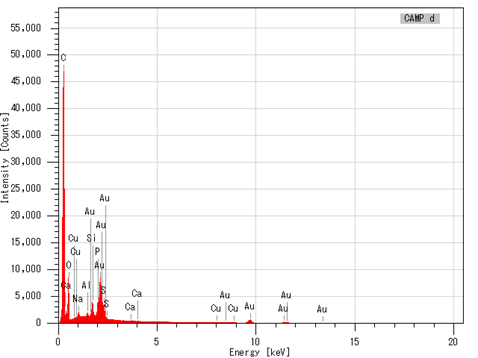 |
| **Figure S13**: EDS of hFOB 1.19 Seeded on a) CA and b) CA-MP Fiber Mats |
